# A nonrandomized controlled trial of individualized exercise prescription combined with remote exercise management in patients who are overweight or obese

**DOI:** 10.1186/s13102-022-00479-1

**Published:** 2022-06-02

**Authors:** Rui Guan, Haijing Li, Yang Jiao, Hong Yu

**Affiliations:** Department of Health Management, Beijing Rehabilitation Hospital, Capital Medical University, Xixiazhuang, Badachu, Shijingshan District, Beijing, 100144 China

**Keywords:** Weight management, Exercise intervention, Body composition, Metabolic indexes

## Abstract

**Background:**

Between 2012 and 2020, the obesity rate increased among Chinese people aged 18 years and above, along with blood pressure, fasting blood glucose, serum total cholesterol, and triglycerides.

**Purpose:**

Our objective was to compare the effects of a combined intervention, including individualized exercise prescription plus remote management versus individualized exercise prescription only, on cardiovascular risk factors in patients who are overweight or obese, with the aim of establishing a more effective remote model of health management than self-management.

**Methods:**

This nonrandomized controlled trial (ChiCTR2100046307) studied patients who are overweight or obese at model labor health management centers from January 2019 to December 2019, including 55 people in the experimental group and 34 in the control group. The relevant indexes of all the research objects from both experimental group and control group were examined. Participants in the experimental group were given individualized exercise prescription combined with remote exercise management over a period of 3 months. The control group was prescribed exercise only at time of enrollment and taught about exercise once, followed by voluntary exercise and self-management for 3 months.

**Result:**

After adjusting for baseline differences, the changes in weight (−2.72 ± 4.03 kg versus 0.32 ± 2.50 kg, *P* < 0.0001), body mass index (−0.99 ± 1.44 kg/m^2^ versus 0.11 ± 0.92 kg/m^2^, *P* < 0.0001), waist circumference (−2.98 ± 6.29 cm versus 0.60 ± 5.33 cm, *P* < 0.0001), visceral fat area (−9.75 ± 19.68 cm^2^ versus −1.31 ± 12.37 cm^2^, *P* = 0.028), body fat (− 2.65 ± 3.52 kg versus 0.54 ± 2.67 kg, *P* < 0.0001), body fat rate (−2.50 ± 3.32% versus 0.21 ± 3.30%, *P* < 0.0001), uric acid (−9.75 ± 19.68 µmol/L versus −1.31 ± 12.37 µmol/L, *P* = 0.028), serum total cholesterol (−0.11 ± 0.40 mmol/L versus −0.11 ± 0.59 mmol/L, *P* = 0.004), fasting insulin (− 2.36 ± 5.20 μU/mL versus 1.22 ± 7.34 μU/mL, *P* = 0.009), and homeostatic model assessment of insulin resistance (−0.62 ± 1.25 versus 0.14 ± 1.83, *P* = 0.022) were significantly better in the experimental group than in the control group after intervention.

**Conclusion:**

Individualized exercise prescription combined with remote management in patients who are obese or overweight facilitated weight and fat loss, lowered blood pressure and serum total cholesterol, improved glucose metabolism and insulin resistance, and reduced cardiovascular risk factors. The intervention was superior to conventional education in terms of weight loss, fat reduction, total cholesterol reduction, fasting insulin reduction, and amelioration of insulin resistance.

## Background

Obesity has become a global public health concern [1]. The obesity rate among Chinese residents aged 18 years and older increased from 11.9% in 2012 to 16.4% in 2020, while blood pressure, blood glucose, serum total cholesterol (TC), and triglycerides (TG) also showed an increasing trend [2, 3]. In addition to increasing body mass index (BMI), obesity may affect the heart through its effect on known risk factors, including dyslipidemia, hypertension, and glucose intolerance, in addition to as-yet unrecognized mechanisms [4].

Visceral fat, an important component of the body, is also an independent risk factor for cardiovascular events in patients with obesity that is highly positively correlated with the incidence of cardiovascular diseases and various metabolic diseases [5]. Multiple studies have shown that overweight and obesity increase the risk of cardiovascular events [6–8]. Therefore, reducing the risk of cardiovascular disease in people who are overweight/obese through a variety of interventions is of great significance for the prevention and treatment of cardiovascular disease.

Physical inactivity is now regarded as one of the most serious global public health issues. Remote physical activity (PA) promotion programs should be effective if they are individually personalized and involve habit modification approaches, personal coaching, and regular reminders. Physical inactivity has been connected to technical advances, automation, increased use of motorized transportation, and a rise in sedentary leisure-time activities [9]. The main self-reported impediments to leisure time PA in working-aged individuals are a lack of time and motivation [10]. Adults require assistance in overcoming personal barriers in order to achieve PA-related behavioral changes. In recent years, there has been an increase in interest in therapies offered without a face-to-face connection. Mobile phone and web-based communication techniques are widely available and allow access to a large demographic [11]. Remotely administered programs are thought to be more cost-effective than face-to-face interventions. Another advantage is the elimination of time, transit, and personal interaction obstacles (i.e., fear of prejudice) [12]. Telephone contact to provide feedback or to promote behavior changes was most successful for increasing self-reported PA in remote interventions [11].

Changing lifestyle through increasing physical activity is an important part of cardiovascular risk factor management measures. The effects of exercise on weight loss and management of cardiovascular risk factors vary greatly among individuals, which can be reflected in demographic characteristics [13], training program characteristics [14], and other aspects. Evidence suggests that aerobic exercise programs can also improve body composition (weight/fat loss with muscle preservation) and physical function among individuals with obesity [15]. Therefore, for overweight/obese individuals, appropriate individualized exercise prescriptions need to be developed on the basis of their degree of overweight and obesity, comorbidities, age, gender, exercise capacity, lifestyle, diet and exercise habits, social and family status, and other factors. The weight loss effect and health benefits are considered along with the executability and exercise adherence of the exercise program to achieve the desired weight loss effect and to reduce the risk of cardiovascular disease from various aspects such as weight, body fat, and metabolic index [16].

Weight management based on exercise intervention requires effective exercise monitoring and long-term follow-up. The effectiveness of exercise management can be compromised if patients are not effectively monitored and managed during their discharge from the hospital. A meta-analysis [17] found that additional exercise on admission mostly benefited patients who were not independently mobile. However, most of the exercise management measures implemented outside the hospital adopt the means of publicity and education and rely on patients’ conscious behavior, failing to achieve remote active management and unable to fully grasp their actual exercise situation. Therefore, the exercise management of patients who are overweight/obese should focus on out-of-hospital management, while the monitoring of exercise-related data of patients (such as exercise intensity, time, frequency, etc.) is inevitably inseparable from remote monitoring technology. Especially during the coronavirus disease 2019 (COVID-19) pandemic, the implementation of remote sports management for patients who are overweight/obese can effectively reduce the risk due to the epidemic by avoiding the gathering of people. Exploring effective models of remote sports management can provide similar health management measures suitable for public health emergency response and meet the health management requirements of patients in special situations [18].

In the face of the severe situation of overweight/obesity in China, it is urgent to develop exercise intervention technology for people who are overweight/obese and examine the suitability of exercise intervention programs for Chinese people. The purpose of this study was to compare the effects of individualized exercise prescriptions plus remote exercise management with individualized exercise prescriptions alone on risk factors for obesity cardiovascular disease, and to provide a theoretical basis for developing appropriate interventions and creating a more effective health management reference model than self-management.

## Methods

### Participants

Patients who were overweight or obese were selected from the Model Worker Health Management Center of Beijing Rehabilitation Center affiliated to Capital Medical University from January 2019 to December 2019, and 89 patients were selected as study participants according to the principle of voluntary registration and strict screening. The definition of overweight and obesity was adopted according to expert Consensus and Group Standards on Weight Management of Overweight or Obese people [19]: 24 kg/m^2^ ≤ BMI < 28 kg/m^2^ for overweight; BMI ≥ 28 kg/m^2^ for obesity.

Inclusion criteria [20]: (1) 18–65 years old, male or female; (2) BMI ≥ 24 kg/m^2^, stable weight in recent 3 months, sedentary lifestyle; (3) no exercise habit and no other exercise plan; (4) not dieting to lose weight; (5) not taking any medications that may affect weight; (6) informed consent and acceptance of the test. Exclusion criteria: (1) secondary obesity or medicated obesity; (2) BMI ≥ 40 kg/m^2^; (3) unable or unwilling to sign the informed consent; (4) patients with clearly diagnosed cardiovascular and cerebrovascular diseases (including myocardial infarction, heart failure, atrial fibrillation, atrial flutter, stroke, etc.); (5) having a clearly diagnosed malignant tumor; (6) mental illness and cognitive impairment; (7) persons with severe physical disabilities who cannot be examined; (8) patients with severe arrhythmia or Brugada syndrome; (9) pregnant and lactating women; (10) those who do not have the ability or conditions to perform the exercise according to the test plan; (11) those who cannot return to the hospital for review after the intervention; (12) unable to obtain exercise heart rate data.

### Study design

**Fig. 1:**
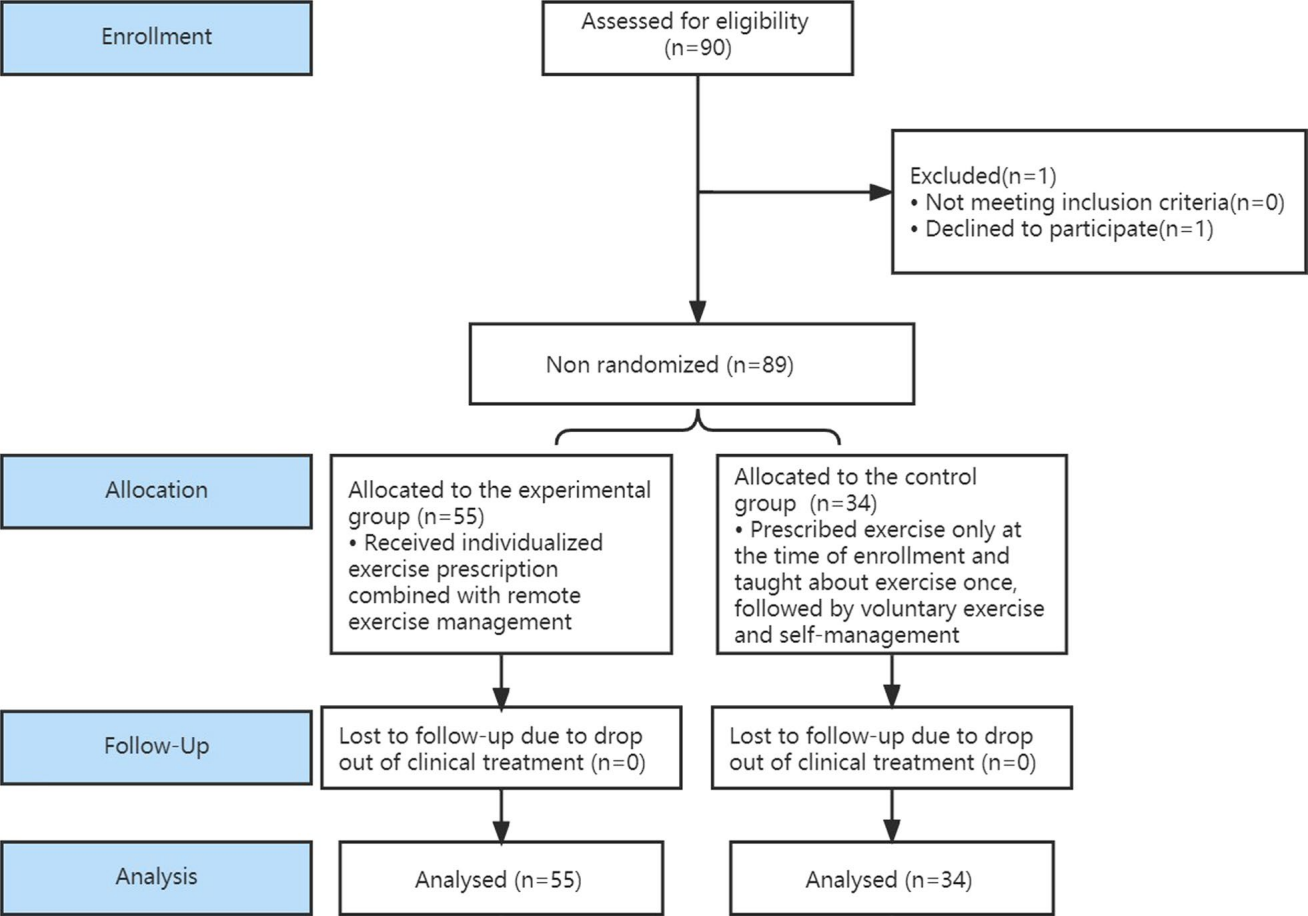
Flow chart of included participants

This study was a nonrandomized controlled trial (clinical trial register number ChiCTR2100046307, first registration on 12 May 2021) including 55 people in the experimental group and 34 in the control group. The study was approved by the ethics committee of our hospital. All the participants were recruited and grouped according to the participants’ willingness, until the sample size was sufficient and the recruitment period ended. No one dropped out of the trial. A flow chart of the study design is shown in Fig. 1. All individuals in the experimental group and control group were examined for relevant indicators: height, weight, BMI, waist circumference (WC), hip circumference (HC), blood pressure (BP), the biochemical indexes of blood [fasting blood glucose (FBG), hemoglobin a1c (HbA1c), fasting insulin (FI), uric acid (UA), TG, TC, low-density lipoprotein cholesterol (LDL-C), and high-density lipoprotein cholesterol (HDL-C)], and homeostatic model assessment of insulin resistance (HOMA-IR; FBG (mmol/L) × FI (mU/L)/22.5). Body fat (BF), skeletal muscle content (SMC), and BF percentage were measured by a human composition analyzer (InBody720, Biospace, Korea) [21]. Visceral fat area (VFA) was measured by a visceral fat analyzer (HDS-2000, Omron, Japan) [22].

The participants in the experimental group underwent individualized exercise prescription combined with remote management for a period of 3 months. The control group, on the other hand, received only an individualized exercise prescription. Using the American College of Sports Medicine (ACSM) guidelines for exercise testing and prescription, the BH-QS-100 system (Beijing Flash and Health Technology Co., Ltd., Beijing) was used to help the participants achieve their physical activity goals. The BH-QS-100 system monitors the participants’ reserve heart rate (HR), ejection time fraction, left heart load, and myocardial perfusion index and health status (including myocardial viability, as well as systolic, diastolic, pulse, and central artery systolic pressure and growth index status to assess arterial elasticity, and individual speed and incline settings for the running platform), thereby providing feedback to maintain the participants’ moderate-intensity aerobic exercise level in a safe and effective range [23–26].

The specific procedures are as follows. (1) Exercise prescriptions were provided to all participants using an exercise and health risk assessment system, the BH-QS-100, which combined information on gender, age, medical history, cardiovascular function, and cardiopulmonary endurance sports of the study participants to set an effective heart rate range achieved by aerobic exercise (below the lower limit of effective heart rate, insufficient exercise intensity and low exercise efficiency; above the upper limit of effective heart rate, prone to sports injury and exercise risk). (2) Remote management was performed through a MioFuse exercise heart rate watch (Physi-Cal, USA) connected to a smartphone application via Bluetooth to monitor the experimental group in order to assess the exercise intensity of the patients, and monitor and guide the exercise heart rate. The function of heart rate monitoring was activated during exercise, and the curve of heart rate change during exercise was recorded by the watch. Heart rate during aerobic exercise should be controlled within the effective heart rate range prescribed. After exercise, the exercise data in the watch were uploaded to the Health Management Station (BH-HMW-100) through the mobile phone software. (3) At the health management workstation, project team members could view the exercise status of the research object, and supervise, guide, and provide feedback on the exercise status. (4) The exercise intervention plan for the experimental group was as follows: aerobic exercise one time every other day, with an effective exercise time of 40 min each time; resistance exercise (10 pushups per set, 2 sets; 6 situps per set, 2 sets; 18 squats per set, 2 sets) two times per week. Effective exercise refers to exercise in which the heart rate falls within the effective heart rate range prescribed by exercise prescription [23, 27].

In the control group, participants were prescribed the same exercise intervention program as in the experimental group (only one exercise-related education session was given at the time of enrollment), and then were asked to self-manage through self-directed exercise (without remote monitoring and guidance of exercise) for 3 months.

In terms of diet, participants in the experimental group and control group were asked to record their daily diet for the first 3 months of the trial and provide feedback to ensure that there was no significant change in diet compared with the pretrial period. Protein, carbohydrate, and fat in the participants’ equilibrium diet accounted for about 15%, 60%, and 25% of total energy, respectively. Estimated energy requirements (EER, kcal per day) were calculated as EER = 662 − (9.53 × age [years]) + PAL (level of physical activity) × {(15.91 × weight [kg]) + (539.6 × height [m])} for males and EER = 354 − (6.91 × age [years]) + PAL × {(9.36 × weight [kg]) + (726 × height [m])} for females [28]. Compliance was defined as completing at least 90% of the exercise sessions in both groups. Participants who were noncompliant for the trial would be excluded from the research. All participants followed the study’s procedure and completed all the evaluations. After 3 months, all individuals in the experimental group and control group were reviewed for all relevant indicators.

## Statistical analysis

SPSS 22.0 was used for statistical analysis. Measurement data are expressed as mean ± standard deviation (*x* ± *s*). Paired sample *t*-test or rank-sum test were used for comparison of observation indexes before and after intervention in the groups. The effects of individual exercise prescription combined with remote exercise management on cardiovascular risk factors were evaluated by the delta indicators before and after the intervention. The delta indicators were the changes in the absolute values (subtracting *t*_−1_ from *t*_0_); the percentage values were calculated as [(*t*_0_/*t*_−1_) × 100 − 100]. Enumeration data are expressed as constituent ratio or rate (%), and comparison between the two groups was performed by chi-square test. In determining the required sample size to achieve 1 − *β* = 0.80 and *α* = 0.05, a two-sample *t*-test was conducted, and the statistical power results indicated that a minimum of 34 participants were required in each group. *P* < 0.05 was considered statistically significant.

## Results

### Basic characteristics of the two groups

A total of 55 participants (23 males and 32 females) were recruited in the experimental group with an average age of 33.84 ± 7.91 years. A total of 34 participants (9 males and 25 females) were recruited in the control group with an average age of 33.94 ± 8.69 years. There was no significant difference between the two groups in terms of gender, age, or other baseline characteristics (Table 1). Both the experimental group and control group had complete data records and no missing data.

**Table 1:** Comparison of the baseline characteristics between the two groups

	Experimental group (*n* = 55) Before intervention	Control group (*n* = 34) Before intervention	*t*	*P*
Weight (kg)	78.54 ± 12.58	75.29 ± 12.71	−1.180	0.241
BMI (kg/m^2^)	27.86 ± 3.17	27.59 ± 2.81	−0.399	0.691
WC (cm)	89.27 ± 7.85	88.66 ± 9.12	−0.355	0.738
HC (cm)	101.36 ± 5.73	101.19 ± 6.89	−0.128	0.899
SBP (mmHg)	127.20 ± 16.60	124.85 ± 15.50	−0.664	0.504
DBP(mmHg)	77.13 ± 10.78	76.97 ± 13.28	−0.061	0.952
VC (cm^2^)	83.31 ± 32.82	80.43 ± 33.49	−0.399	0.691
BF (kg)	26.39 ± 6.45	26.78 ± 5.04	0.299	0.706
SMC (kg)	28.99 ± 6.27	27.11 ± 6.28	−1.380	0.171
BF (%)	33.55 ± 6.34	35.61 ± 5.14	1.594	0.114
UA (µmol/L)	361.96 ± 96.55	322.12 ± 90.45	−1.937	0.056
TC (mmol/L)	4.52 ± 0.71	4.76 ± 0.81	1.495	0.139
TG (mmol/L)	1.37 ± 0.65	1.57 ± 1.42	0.919	0.361
LDL-C (mmol/L)	1.20 ± 0.19	1.29 ± 0.21	1.865	0.066
HDL-C (mmol/L)	2.77 ± 0.67	2.87 ± 0.66	0.752	0.454
HbA1c (%)	5.60 ± 0.45	5.58 ± 0.38	−0.184	0.855
FBG (mmol/L)	5.21 ± 0.62	5.07 ± 0.47	−1.129	0.262
FI (μU/mL)	11.16 ± 5.34	11.45 ± 5.52	0.247	0.805
HOMA-IR	2.59 ± 1.40	2.58 ± 1.25	−0.027	0.978

*WC* waist circumference, *HC* hip circumference, *SBP* systolic blood pressure, *DBP* diastolic blood pressure, *VFA* visceral fat area, *BF* body fat, *SMC* skeletal muscle content, *UA* uric acid, *TC* total cholesterol, *TG* triglycerides, *LDL-C* low-density lipoprotein cholesterol, *HDL-C* high-density lipoprotein cholesterol, *HbA1c* hemoglobin A1c, *FBG* fasting blood glucose, *FI* fasting insulin, *HOMA-IR* homeostatic model assessment of insulin resistance

### Comparison of general conditions of the participants in the two groups before and after intervention

After adjusting for baseline differences (Table 2, delta indicator), the changes in weight, BMI, and WC were significantly lower in the experimental group than in the control group after intervention (all *P* < 0.01).

**Table 2:** Comparison of the delta indicators between the two groups

	Experimental group (*n* = 55)			Control group (*n* = 34)			For delta indicators	
	Before intervention *t*_−1_	After intervention *t*_0_	Delta changes	Before intervention *t*_−1_	After intervention *t*_0_	Delta changes	*t*	*P*
Weight (kg)	78.54 ± 12.58	75.82 ± 12.58	−2.72 ± 4.03	75.29 ± 12.71*	75.61 ± 13.34*	0.32 ± 2.50	3.946	0.000
BMI (kg/m^2^)	27.86 ± 3.17	26.87 ± 3.45	−0.99 ± 1.44	27.59 ± 2.81	27.70 ± 3.05	0.11 ± 0.92	3.950	0.000
WC (cm)	89.27 ± 7.85	86.29 ± 9.79	−2.98 ± 6.29	88.66 ± 9.12*	89.26 ± 10.29*	0.60 ± 5.33	2.764	0.000
HC (cm)	101.36 ± 5.73	98.73 ± 7.33	−2.64 ± 6.66	101.19 ± 6.89	101.35 ± 6.57	0.16 ± 6.58	1.935	0.056
SBP (mmHg)	127.20 ± 16.60	122.44 ± 15.82	−4.76 ± 14.52	124.85 ± 15.50	123.88 ± 13.00	−0.97 ± 12.13	1.237	0.207
DBP (mmHg)	77.13 ± 10.78	72.07 ± 11.66	−5.05 ± 10.04	76.97 ± 13.28	75.26 ± 10.74	−1.71 ± 9.79	1.544	0.126

*WC* waist circumference, *HC* hip circumference, *SBP* systolic blood pressure

*The control group was prescribed exercise on admission only and taught exercise once, followed by voluntary exercise and self-management for 3 months without remote monitoring and guided exercise

### Comparison of body composition between the two groups before and after intervention

After adjusting the baseline differences (Table 3, delta indicator), the changes in VFA, BF, and BF% were significantly lower in the experimental group than in the control group after intervention (all *P* < 0.05).

**Table 3:** Comparison of body composition between the two groups

	Experimental group (*n* = 55)			Control group (*n* = 34)			For delta indicators	
	Before intervention *t*_−1_	After intervention *t*_0_	Delta changes	Before intervention *t*_−1_	After intervention *t*_0_	Delta changes	*t*	*P*
VFA (cm^2^)	83.31 ± 32.82	73.55 ± 30.60	−9.75 ± 19.68	80.43 ± 33.49	79.11 ± 31.83	−1.31 ± 12.37	2.239	0.028
BF (kg)	26.39 ± 6.45	23.74 ± 7.19	−2.65 ± 3.52	26.78 ± 5.04*	27.32 ± 6.17*	0.54 ± 2.67	4.533	0.000
SMC (kg)	28.99 ± 6.27	29.25 ± 6.12	0.26 ± 1.74	27.11 ± 6.28*	27.01 ± 6.31*	−0.10 ± 1.99	−0.881	0.381
BF (%)	33.55 ± 6.34	31.05 ± 7.12	−2.50 ± 3.32	35.61 ± 5.14*	35.81 ± 6.08*	0.21 ± 3.30	3.749	0.000

*VFA* visceral fat area, *BF* body fat, *SMC* skeletal muscle content

*The control group was prescribed exercise on admission only and taught exercise once, followed by voluntary exercise and self-management for 3 months without remote monitoring and guided exercise

Further analysis showed (Table 4, delta indicators) that, after the intervention, BF and BF% were significantly lower in the experimental group than in the control group (all *P* < 0.05).

**Table 4:** Comparison of body composition between the two groups by gender

	Men in experimental group (*n* = 23)			Men in control group (*n* = 9)			*P*	Women in experimental group (*n* = 32)			Women in control group (*n* = 25)			*P*
	*t*_−1_	*t*_0_	^Δ^changes	*t*_−1_	*t*_0_	^Δ^changes		*t*_−1_	*t*_0_	^Δ^changes	*t*_−1_	*t*_0_	^Δ^changes	
VFA (cm^2^)	95.83 ± 30.36	79.61 ± 31.11	−16.22 ± 23.37	108.13 ± 34.30	103.58 ± 36.77	−4.56 ± 15.04	0.18	74.31 ± 31.97	69.2 ± 29.96	−5.11 ± 15.29	70.45 ± 27.54	70.3 ± 25.24	−0.15 ± 11.38	0.18
BF (kg)	25.33 ± 7.16	21.79 ± 7.35	−3.54 ± 3.37	27.21 ± 5.93	28.8 ± 8.3	1.59 ± 3.76	0.00	27.15 ± 5.88	25.15 ± 6.85	−2.01 ± 3.53	26.62 ± 4.81	26.79 ± 5.32	0.16 ± 2.14	0.01
SMC (kg)	34.17 ± 6.29	34.92 ± 5.04	0.75 ± 2.46	36.44 ± 3.19	35.72 ± 5.16	−0.72 ± 3.54	0.19	25.27 ± 2.48	25.18 ± 2.5	−0.1 ± 0.81	23.74 ± 2.52	23.87 ± 2.66	0.13 ± 1.04	0.36
BF (%)	28.92 ± 5.70	25.68 ± 5.67	−3.25 ± 3.12	29.5 ± 4.00	31.13 ± 7.98	1.63 ± 5.08	0.00	36.88 ± 4.42	34.91 ± 5.54	−1.97 ± 3.41	37.81 ± 3.47	37.5 ± 4.28	−0.31 ± 2.31	0.04

*t*_−1_ before intervention, *t*_0_ after intervention, ^*Δ*^*changes* delta changes, *VFA* visceral fat area, *BF* body fat, *SMC* skeletal muscle content

### Comparison of metabolic indexes between the two groups before and after intervention

After adjusting for baseline differences (Table 5, delta indicator), the changes in UA, TC, FI, and HOMA-IR were significantly lower in the experimental group than in the group control group after intervention (all *P* < 0.05).

**Table 5:** Comparison of metabolic indexes between the two groups

	Experimental group (*n* = 55)			Control group (*n* = 34)			For delta indicators	
	Before intervention *t*_−1_	After intervention *t*_0_	Delta changes	Before intervention *t*_−1_	After intervention *t*_0_	Delta changes	*t*	*P*
UA (μmol/L)	361.96 ± 96.55	357.56 ± 103.13	−9.75 ± 19.68	322.12 ± 90.45	307.96 ± 91.46	−1.31 ± 12.37	2.239	0.028
TC (mmol/L)	4.52 ± 0.71	4.40 ± 0.72	−0.11 ± 0.40	4.76 ± 0.81	4.95 ± 0.92	−0.11 ± 0.59	2.953	0.004
TG (mmol/L)	1.37 ± 0.65	1.31 ± 0.67	−0.07 ± 0.64	1.57 ± 1.42	1.47 ± 1.15	−0.11 ± 0.59	−0.319	0.750
LDL-C (mmol/L)	1.20 ± 0.19	1.23 ± 0.21	0.02 ± 0.13	1.29 ± 0.21	1.32 ± 0.24	0.04 ± 0.23	0.308	0.705
HDL-C (mmol/L)	2.77 ± 0.67	2.79 ± 0.68	0.03 ± 0.39	2.87 ± 0.66	3.01 ± 0.72	0.14 ± 0.40	1.286	0.202
HbA1c (%)	5.60 ± 0.45	5.63 ± 0.45	0.03 ± 0.37	5.58 ± 0.38	5.49 ± 0.36	−0.09 ± 0.26	−1.701	0.092
FBG (mmol/L)	5.21 ± 0.62	4.92 ± 0.5	−0.28 ± 0.52	5.07 ± 0.47	4.94 ± 0.74	−0.13 ± 0.69	1.229	0.222
FI (μU/mL)	11.16 ± 5.34	8.80 ± 5.60	−2.36 ± 5.20	11.45 ± 5.52	12.67 ± 7.59	1.22 ± 7.34	2.688	0.009
HOMA-IR	2.59 ± 1.40	1.97 ± 1.33	−0.62 ± 1.25	2.58 ± 1.25	2.72 ± 1.61	0.14 ± 1.83	2.327	0.022

*UA* uric acid, *TC* total cholesterol, *TG* triglycerides, *LDL-C* low-density lipoprotein cholesterol, *HDL-C* high-density lipoprotein cholesterol, *HbA1c* hemoglobin A1c, *FBG* fasting blood glucose, *FI* fasting insulin, *HOMA-IR* homeostatic model assessment of insulin resistance

Statistical tests of delta changes showed that remote management (experimental group) was superior to self-management (control group) in improving weight, BMI, WC, VFA, BF, UA, TC, FI, and HOMA-IR.

## Discussion

Obesity is an independent risk factor for cardiovascular disease. In addition, obesity causes insulin resistance and endothelial dysfunction due to metabolites formed by lipids, hormones, and pro-inflammatory cytokines [29, 30]. Endothelial dysfunction is associated with cardiovascular diseases, such as atherosclerosis, hypertension, hyperlipidemia, and insulin resistance, which alter insulin signaling pathways [31]. Compared with adults with a BMI of 25 kg/m^2^, the incidence of cardiovascular disease in adults with a BMI greater than 30 kg/m^2^ was significantly increased, and the mortality from cardiovascular disease increased by 40% for every 5 kg/m^2^ increase in BMI [32]. A Chinese cohort study shows that maintaining BMI < 25.0 kg/m^2^ can reduce the incidence of cardiovascular disease by 5.0% in adults [33].

In this study, there were statistically significant differences in body weight and BMI in the experimental intragroup, specifically reflected in the significant decrease in body weight and BMI in the experimental group after the intervention compared with before the intervention. There were also significant differences in body weight and BMI between groups in delta changes. Compared with the control group, the experimental group showed a significant decrease in body weight and BMI, suggesting that individualized exercise prescription combined with remote exercise management can help obese and overweight individuals lose weight and thus reduce the risk of cardiovascular disease, and that the intervention effect is better than conventional education.

Obesity is also an important risk factor for high blood pressure. For every 5% increase in body weight, individuals with normal blood pressure experience a 20–30% increase in their risk of hypertension in the future, and for every 20 mmHg or 10 mmHg increase in systolic blood pressure (SBP), the risk of cardiovascular and cerebrovascular complications doubles. Controlling BP can reduce the risk of cardiovascular diseases. For every 10 mmHg reduction in SBP, the incidence of coronary heart disease decreases by 17% [34].

In this study, there were significant intragroup differences in SBP and diastolic blood pressure (DBP) in the experimental group, which showed that blood pressure was significantly lower after the intervention than before the intervention, suggesting that individualized exercise prescription combined with remote exercise management can help people who are obese or overweight reduce the risk of cardiovascular disease by improving BP. There were significant intragroup differences in BF content, BF percentage, and VFA in the experimental group, specifically reflected in the significant reduction of these indicators in the experimental group after the intervention compared with before the intervention. There were also statistical differences in delta changes of BF content, BF percentage, and VFA between groups, and the degree of reduction in these indicators was significantly higher in the experimental group than in the control group, indicating that individualized exercise prescription combined with remote exercise management can not only reduce the content of fat in the body of people who are obese or overweight, but also affect the distribution of fat in the body, and the intervention is more effective than conventional education. The level of adipose tissue, especially visceral fat, is closely related to the occurrence of cardiovascular disease risk factors [35, 36]. The indisputable link between visceral adipose tissue and cardiometabolic risk also makes it a major target for lifestyle strategy-based health risk management [37]. Body fat distribution is an important risk factor for obesity-related diseases, such as cardiovascular disease [38]. Exercise significantly reduces visceral fat and cardiovascular disease risk, which is associated with exercise-induced negative energy balance [39, 40]. However, body composition differs between men and women, with women having proportionally more fat mass and men more muscle mass. Although men and women are both susceptible to obesity, health consequences differ between the sexes [41]. Therefore, we further analyzed the body composition data of the two gender groups (Table 4), and the results showed that BF content and BF percentage in the experimental group still showed significant improvements over the control group, but VFA did not. The delta changes of VFA showed a larger standard deviation, indicating that we need more samples or gender–age subgroups to test body composition in the future.

In terms of blood glucose metabolism, this study found that, after 3 months of remote management of aerobic exercise combined with resistance exercise, FBG, FI, and HOMA-IR were significantly reduced in the experimental group, which was similar to the results of Kim et al. [42]. Glucose/insulin metabolism markers are one of the biomarkers of cardiac metabolism [43]. Metabolic diseases involving insulin resistance are becoming a major risk factor for cardiovascular disease. Clinically, hypertension, obesity, and cardiovascular diseases often have insulin resistance as their core, accompanied by numerous functional and structural cardiovascular abnormalities such as vascular endothelial cell dysfunction that coexist and promote each other [44, 45]. Exercise can reduce blood sugar, which may be due to the fact that aerobic exercise can increase energy consumption, increase the activity of aerobic metabolic enzymes in muscle cells, enhance glucose metabolism, promote the absorption of sugar by cells, increase the speed of sugar transfer to cells, improve the process of sugar decomposition and utilization, and reduce blood sugar levels [46]. At the same time, aerobic exercise can enhance the ability of insulin receptors to bind to insulin in skeletal muscle cell membrane and liver cell membrane, and improve the sensitivity of peripheral tissues to insulin [47]. Exercise interventions in overweight/obese individuals can reduce blood sugar levels and improve insulin sensitivity, thereby reducing the risk of cardiovascular disease. This study found no significant effect of individualized exercise prescription with combined with remote management intervention on glycated hemoglobin, probably because glycated hemoglobin usually reflects the glycemic control after 8–12 weeks, while the duration of intervention in this study was short and the differences in glycated hemoglobin within and between groups were not fully evident.

It has been established that hypercholesterolemia is an independent risk factor for cardiovascular disease. Management of hypercholesterolemia is fundamental to all prevention strategies, both primary and secondary, at all stages of cardiovascular risk. It was found that the serum TC level was positively correlated with the incidence of cardiovascular disease [48–50]. Therefore, lowering TC levels can control the risk of cardiovascular disease. In the present study, TC levels were significantly lower in the experimental group after the intervention than before the intervention. The post-intervention difference was significant between the two groups, suggesting that individualized exercise prescription combined with remotely managed exercise can help reduce serum TC levels and, thus, reduce the risk of cardiovascular disease. Exercise reduces the production of fat and TC by promoting the whole continuous process of oxidative decomposition and energy utilization of the tricarboxylic acid cycle of fatty acids [51] and is an effective measure to reduce cholesterol and control cardiovascular risk factors. Meanwhile, we found that the intervention of individualized exercise prescription combined with remote management had no short-term effects on TG, LDL-C, HDL-C, or UA.

One limitation of this study is its small sample size. Second, we could only monitor aerobic exercise with an exercise heart rate wristwatch and did not track resistance exercise because there was no objective basis to demonstrate the participants’ performance, which would have had some impact on the participants’ resistance exercise test results. Third, InBody’s fat mass assessment is based on the principle of bioelectrical impedance (BIA), which works well in assessing fat mass or change but has limited ability to measure visceral fat [52]. Finally, further evaluation of the long-term sustainability and effectiveness of the program will need to be conducted by different institutions and over a longer period of time. In addition, data on endurance exercise, dietary prescription, and intake monitoring need to be obtained in future studies.

## Conclusion

As individualized exercise prescription combined with remote exercise management interventions can help people who are overweight/obese lose weight, reduce fat, lower BP and TC, improve glucose metabolism, and ameliorate insulin resistance, it is an effective health management measure and nonpharmacological treatment for overweight and obesity. Individualized exercise prescription combined with remote exercise management, as an appropriate technique for exercise management of people who are overweight/obese, can effectively control cardiovascular risk factors, and is worthy of promotion and application.

## Data Availability

The data supporting the findings of this study are available from the corresponding author on reasonable request.
